# Role of gut microbiota in identification of novel TCM-derived active metabolites

**DOI:** 10.1007/s13238-020-00784-w

**Published:** 2020-09-15

**Authors:** Tzu-Lung Lin, Chia-Chen Lu, Wei-Fan Lai, Ting-Shu Wu, Jang-Jih Lu, Young-Mao Chen, Chi-Meng Tzeng, Hong-Tao Liu, Hong Wei, Hsin-Chih Lai

**Affiliations:** 1 Department of Medical Biotechnology and Laboratory Science, College of Medicine, Chang Gung University, Gueishan, Taoyuan, 33302 Taiwan China; 2 Department of Respiratory Therapy, Fu Jen Catholic University, New Taipei City, 24205 Taiwan China; 3 Department of Chest Medicine, Internal Medicine, Fu Jen Catholic University Hospital, Fu Jen Catholic University, New Taipei City, 24205 Taiwan China; 4 Department of Medicine, Chang Gung University, Taoyuan, 33302 Taiwan China; 5 Department of Laboratory Medicine and Internal Medicine, Linkou Chang Gung Memorial Hospital, Taoyuan, 33305 Taiwan China; 6 Central Research Laboratory, Xiamen Chang Gung Hospital, Xiamen, 361026 China; 7 Bachelor Degree Program in Marine Biotechnology, College of Life Sciences, National Taiwan Ocean University, Keelung, 20224 Taiwan China; 8 School of Pharmaceutical Sciences, Xiamen University, Xiamen, 361005 China; 9 College of Basic Medicine, Hubei University of Chinese Medicine, Wuhan, 430065 China; 10 Central Laboratory, Clinical Medicine Scientific and Technical Innovation Park, Shanghai Tenth People’s Hospital, Tongji University, Shanghai, 200435 China; 11 Microbiota Research Center and Emerging Viral Infections Research Center, Chang Gung University, Taoyuan, 33302 Taiwan China; 12 Research Center for Chinese Herbal Medicine and Research Center for Food and Cosmetic Safety, College of Human Ecology, Chang Gung University of Science and Technology, Gueishan, Taoyuan, 33303 Taiwan China

**Keywords:** Traditional Chinese Medicine, herbs, microbiota, transformation, multiomics

## Abstract

Traditional Chinese Medicine (TCM) has been extensively used to ameliorate diseases in Asia for over thousands of years. However, owing to a lack of formal scientific validation, the absence of information regarding the mechanisms underlying TCMs restricts their application. After oral administration, TCM herbal ingredients frequently are not directly absorbed by the host, but rather enter the intestine to be transformed by gut microbiota. The gut microbiota is a microbial community living in animal intestines, and functions to maintain host homeostasis and health. Increasing evidences indicate that TCM herbs closely affect gut microbiota composition, which is associated with the conversion of herbal components into active metabolites. These may significantly affect the therapeutic activity of TCMs. Microbiota analyses, in conjunction with modern multiomics platforms, can together identify novel functional metabolites and form the basis of future TCM research.

## Introduction

### TCM and herbal formulae

TCM has been used for centuries in China to alleviate symptoms, treat disease, and promote well-being in Chinese patients (Zhao et al., 2014). In contrast to modern Western medicine, research progress made in TCM is often inhibited because of the inherent complexity of herbs as medicine and a comparative lack of modern scientific validation. Accordingly, TCM research must be modernized by meeting the scientific method.

Historically, recordings that emerged from functional TCM herbal ingredients and aimed at treating specific diseases, eventually evolved into the creating of specific formulae. These formulae were further revised and assembled to create the TCM version of the “Materia Medica”. These manuals established a solid basis and references of TCM for clinical treatment. Among these references, the “*Shennong Bencao Jing*” (literally, *Shennong*’s Classic of Materia Medica) (Jin et al., 2013), “*HuangDi NeiJing*” (literally *The Yellow Emperor*’*s Classic of Medicine*) (Ni, 1995), and “*Compendium of* “*Materia Medica*” (Li et al., 2014; Hao and Jiang, 2015; Gao et al., 2016; Ding et al., 2020) were featured. TCM-derived materia medicas have been rapidly developed and created complicated herbal networks for clinical applications. Each materia medica contained many formulae, with each formula comprising a combination of herbal drugs. Among these, complex components including carbohydrates/polysaccharides (PS), proteins/peptides, glycolipids/glycoproteins, lipids, together with their metabolic derivatives such as glycosides, amines, fatty acids, flavonoids, terpenoids, phenols, and alkaloids intimately interacted with each other and modulated biological responses of immune cells and the hosts (Li and Kan, 2017; Yu et al., 2018b; Zhang et al., 2020c). Differential agonistic, compatible, or antagonistic interactions occur among TCM herbal ingredients. For instance, the Fuzheng Huayu (FZHY) is mainly composed of Radix Salvia Miltiorrhizae, Cordyceps, Semen Persicae, Gynostemma Pentaphyllum, Pollen Pini, and Fructus Schisandrae Chinensis. It is widely administered to ameliorate chronic liver diseases and functions through modulation of multiple signaling pathways in a number of organs (Chen et al., 2019). FZHY effectively regulates immune functions, optimizes systematic amino acid metabolism and endocrine function, and reduces portal vein hypertension (Chen et al., 2019). These alterations lead to improved liver function and antifibrotic effects. Additionally, FZHY also has no serious adverse reactions (Chen et al., 2019). Further, Gegen Qinlian decoction (GQD), composed of four herbs: Gegen (*Radix Puerariae*), Huangqin (*Radix Scutellariae*), Huanglian (*Rhizoma Coptidis*) and Gancao (Honey-fried Licorice Root) is frequently used in TCM for alleviation of type 2 diabetes (Xu et al., 2015). The Qushi Huayu Decoction (QHD), made up of Herba Artemisiae capillaris, Rhizoma Polygoni cuspidati, Herba Hyperici Japonici, Rhizoma Curcumae longae, and Gardenia jasminoides ameliorates non-alcoholic fatty liver disease (NAFLD) in patients (Feng et al., 2013).

### Current advances in identification of active components from TCM herbs

The basic principle of scientific exploration in Western medicine has been the discovery of functional compounds and their corresponding targets in specific signaling pathways within cells. To achieve this understanding, standardized phytochemistry, pharmacology, pharmacokinetics (PK, absorption/distribution/metabolism/excretion, ADME), pharmacodynamics (PD, effects/action/mechanism), and toxicology research procedures are performed (Chen et al., 2020). Currently, platforms for high throughput screening of compounds, together with stringent functional and safety validations are used to better understand the mechanisms of action of functional compounds. Concordantly, for the development of novel therapeutic drugs from TCM-derived herbs, a similar approach was established (Martel et al., 2017a; Jiang et al., 2020). Through this method, many active components in TCM herbs were identified. One famous example was the discovery of artemisinin, a plant-derived compound with anti-malaria and anti-cancer functions (Zhang et al., 2007; Carqueijeiro et al., 2019). Other examples included berberine purified from berberis, capsaicin from chili peppers, caffeine from coffee beans, ephedrine from Ephedra, chitosan from mushrooms, genistein from soybeans, celastrol from thunder god vine, epigallocatechin gallate from green tea, glycyrrhizin from licorice roots, quercetin from various plants, and curcumin from turmeric (Martel et al., 2017b). Besides small chemicals, functional polysaccharides (PS) derived from TCM herbs have also been characterized, such as those from *Ganoderma lucidum* mycelium (Chang et al., 2015), *Hirsutella sinensis* mycelium (Chang et al., 2015; Wu et al., 2019), and *Poria cocos* (Sun et al., 2019).

To improve the efficiency of screening for novel functional TCM herbal components, new approaches using modern technology have been explored. For example, a luciferase-based high-throughput screening (HTS) assay has been used to integrate multiple chemical messages derived from effective TCM healing formulae. This pipeline can expedite the active ingredient discovery process by reducing replicated leads (Yu et al., 2019a). Beyond this screen, a TCM System Pharmacology Database and Analysis Platform (TCMSP) had been established. The TCMSP is a systematic pharmacology database which compiles drug discovery results from previous herbal medicine experiments. This database contains pharmacochemistry, ADME and toxicity properties, drug likeness and targets, associated diseases, and interaction networks. Importantly, this database can be used to unravel active components in TCM herbs and their targeted cellular pathways (Ru et al., 2014; Li et al., 2020). Exploration of this database can be combined with other systems, such as Gene Ontology (GO) predictions and Kyoto Encyclopedia of Genes and Genomes (KEGG) pathway enrichment analyses to identify potential ameliorative mechanisms of key molecules (Yu et al., 2019a). Additionally, interactions between active molecules and their predicted target proteins may be further predicted by “molecular docking” and protein-protein interaction networks, which may enhance understanding of underlying potential interactions. Finally, the TCMSP database enables the linking of identified compounds to their corresponding targets/pathways involved in disease amelioration. Therefore, through these analyses, axis of component-target-disease (C-T-D) and the corresponding target-pathway (T-P) networks could be established, to further dissect the active compounds, potential targets, and core pathways in treatment of diseases by a specific TCM formula (Li et al., 2020). Subsequently, LC and/or GC-MS/MS can be used to practically monitor the active ingredients of TCMs. This style of pipeline may provide a new, standardized approach to systematically screen TCM herbal components for treatment of diseases (Li et al., 2020). Accordingly, functional molecules in TCMs can be assessed in the context of heterogeneous cell signaling pathways to predict their effects on diseases at immunological, metabolic, and molecular levels (Zhang et al., 2016; Li and Kan, 2017).

Even though many *in vitro* assay-based screening systems are available for high throughput screening, most purified small chemical components directly derived from herbs still suffer from marginal potency, adverse effects, and low bioavailability in animal or clinical studies (Belcher et al., 2019; Liu et al., 2019a; Teijaro et al., 2019). For example, the compound rhein showed beneficial effects on diabetic nephropathy, which is related to reduced levels of TGF-β_1_, renal fibrosis, metabolism, and oxidative stress status (Hu et al., 2019). However, its adverse effects, such as hepatotoxicity, nephrotoxicity, and embryonic toxicity were also highlighted (Yuan et al., 2016). Further, chemical compounds derived from *Polygonum multiflorum* (also known as Heshouwu) showed ameliorative effects on hair-blackening, liver and kidney-tonifying, anti-aging, as well as neuronal disease treatment (Lin et al., 2015). However, these compounds could induce hepatotoxicity, nephrotoxicity and embryonic toxicity (Lin et al., 2015). Comparatively, the PS purified from TCM herbs showed less toxicity and were frequently modified in the host (Chen et al., 2016). So far the underlying molecular mechanism of PS effects remains poorly understood.

## Gut microbiota maintains intestinal homeostasis and promote health

The gut microbiota is a collection of microbes colonizing the intestine (Lin et al., 2014; Tsai et al., 2019; Zmora et al., 2019). More than 100 trillion (10^14^) microbes inhabit the human gastrointestinal (GI) tract, which included about 10 times more bacterial cells than the number of human cells, and over 100 times the amount of genetic contents (microbiome) in contrast to the human genome (Thursby and Juge, 2017). Bacteria at the number between 10^2^–10^4^ colony-forming units (CFU)/mL are found in the first section of the small intestine, the duodenum. Generally, the Lactobacilli, Streptococci, Veillonellae, Staphylococci, Actinobacilli and yeasts dominate the duodenum and jejunum (Lin et al., 2014). The GI microbiota changes markedly from the duodenum to the ileum, with an according increase in bacterial load, reaching up to 10^6^–10^8^ CFU/mL. In the large intestine, commensal bacteria reach high numbers (10^7^–10^12^ CFU/mL) and are extremely diverse. At the same time, the environment of colon is strictly anaerobic. This means that obligate anaerobes that obtain their energy from fermentation dominate. More than 1,500 bacterial species have been identified in the colon of humans (Lin et al., 2014; Chang et al., 2019). However, a large proportion of the GI microbiota bacteria cannot be easily isolated and cultured *in vitro*, necessitating the use of culturomics (Lagier et al., 2018).

Under homeostasis situation, the intestinal microenvironment provides a barrier to prevent the translocation of pathogens or harmful agents (such as the endotoxin LPS) across the intestinal epithelial cells (IECs) into the surrounding lymphoid system and blood (Boyapati et al., 2016). Gut microbiota bacteria maintain host integrity and regulate many important physiological functions, including homeostasis of energy and metabolism, modification of xenobiotics, modulation of intestinal homeostasis, regulation of immunity and protection against pathogens, and even normal host neuronal behavior and cognitive functions (Clemente et al., 2012; Schachter et al., 2018; Lin et al., 2019).

In aberrant physical, chemical, or biological conditions, such as long-term high fat diet, antibiotic treatment, or increased oxidative stress, the composition of gut microbiota changes, lead to GI dysbiosis and disruption of the intestinal mucosa. This dysbiosis results in a damaged intestinal barrier and increased intestinal permeability. Proinflammatory elements, such as pathogen-associated molecular pattern molecules (PAMPS) (mainly the endotoxin, LPS) or damage-associated molecular pattern molecules (DAMPS) (Tang et al., 2012), are increased in intestines and blood, resulting in both local intestinal injury and systemic chronic inflammation (Yan, 2018; Alexandrov et al., 2019). This “leaky gut” phenomenon is closely related to development of chronic inflammation-related diseases. In a broad sense, the gut microbiota appears to be critical in maintaining host homeostasis and health (Lin et al., 2014; Wang et al., 2017a; Gentile and Weir, 2018).

## Close interaction between tcm herbs and gut microbiota in diseases amelioration

**Figure 1:**
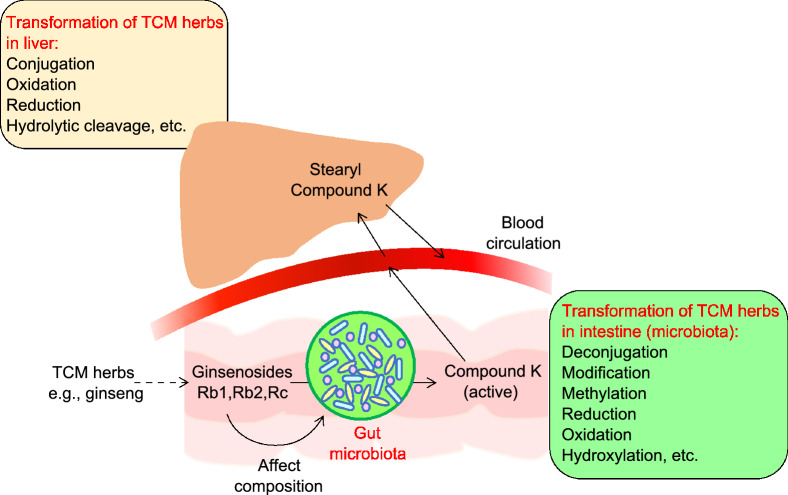
The transformation of **TCM herbal ingredients (ginseng extracts as an example)**. Transformation of TCM herbal ingredients into active metabolites in host was contributed both by gut microbiota and liver. Understanding ginseng’s pharmacokinetics is important for better medication in patients. After oral administration of ginseng, the bioavailability of ginsenosides is low, and the metabolites transformed by gut microbiota may become biologically active. For example, ginsenosides Rb1, Rb2 and Rc were transformed by gut microbiota to form compound K, followed by absorption into the blood (Qi et al., 2011). Compound K showed higher potency and activity compared with ginsenoside Rb1. Compound K adsorbed into blood metabolized again in liver to form stearyl compound K (Kim, 2018). On the other hand, ginsenoside Rb1 also could modulate the composition of gut microbiota (Wan et al., 2017). Therefore, gut microbiota produces active metabolites and plays an important role in the pharmacological action of orally administered ginseng

TCM herbs closely interact with gut microbiota and affect their composition (Peng et al., 2020). Reciprocally, the gut microbiota also plays essential roles in the conversion of carbohydrates, proteins, lipids, and non-nutritive small chemical compounds from TCM herbs into chemical metabolites that may show beneficial or adverse effects on human health (Blaut and Clavel, 2007; Wang et al., 2013; Yu et al., 2018a; Feng et al., 2019; Lu et al., 2019; Qu et al., 2019; Yue et al., 2019; Zhang et al., 2019a, b, 2020c) (Fig. 1 and Table 1). These results indicated that modulation of gut microbiota composition may contribute to the effects of disease amelioration by TCM treatment. For oral treatment, TCM herbs have mostly been prepared by soaking the ingredients in boiling/hot water to generate a water extract that contains a mixture of chemical components, which was generally named as “decoction” (Zhou et al., 2016; Chi et al., 2019; Deng et al., 2019). While some TCM nutraceuticals may directly affect epithelial and immune cells of the digestive tract; others, such as indigestive PS, polyphenols, and alkaloids, etc., may pass through the stomach and reach small and large intestine. Many herbal ingredients are frequently fermented or converted by local gut microbiota to form bioactive, bioavailable, or even toxic metabolites (Lyu et al., 2017; Liu et al., 2018b; Dey, 2019; Wu and Tan, 2019; Yang and Lao, 2019). Depending on use of different formulae, some transformed metabolites may be functionally novel and not clearly defined (Fig. 1). Both changed microbiota bacteria and transformed TCM metabolites may contribute to control of progression of diseases development. Take the TCM effects on amelioration of diabetes as an example: while almost all bacterial phyla seemed to be affected by TCM herbs administration, Bacteroidetes, Firmicutes (and therefore the Firmicutes/Bacteroidetes (F/B) ratio), Proteobacteria, Verrucomicrobia, Cyanobacteria, Deferribacteres, and Actinobacteria were mostly reported (Dey, 2019; Zhang et al., 2019a). Further detailed analyses indicated the abundance of potentially beneficial (such as anti-inflammatory, or SCFAs producers) and harmful (proinflammatory and pathogenic) bacteria could be differentially affected by ingestion of different TCM herbs (Chang et al., 2015; Lyu et al., 2017; Tong et al., 2018; Lin et al., 2019; Nie et al., 2019; Wu et al., 2019). Alteration of the gut microbiota composition is therefore closely related to development of differential immune and metabolic activities in the hosts. Roles of these bacteria on health or disease development are species or even strain dependent under different disease situations, which is under intensive study (Lin et al., 2019).

**Table 1:** Relationship between TCM herbal ingredients, gut microbiota composition, metabolites produced and targeted diseases. ND, not clearly defined.

TCM herbal ingredients	Microbiota affected	Metabolites produced	Target diseases	References
Main component: Decoction or Tang				
Daesi ho Tang (DSHT)	Bacteroidetes, Bacteroidetes/Firmicutes ratio	ND	Obesity	(Hussain et al., 2016)
Gegen Qinlian Decoction (GQD)	*Faecalibacterium prausnitzii*	ND	Type 2 diabetes	(Xu et al., 2015)
Qushi Huayu Decocti (QHD)	Generally affects gut microbiota composition. Does not affect identified Gram-negative bacteria.	ND	NAFLD	(Leng et al., 2020)
Xiexin Tang (XXT)	Adlercreutzia, Alloprevotella, Barnesiella, Ventriosum group, Blautia, Lachnospiraceae UCG-001, Papillibacter, Prevotellaceae NK3B31 group	SCFAs	Type 2 diabetes	(Wei et al., 2018)
Metformin and a specifically designed herbal formula, AMC	Blautia spp., *Faecalibacterium* spp.	ND	Type 2 diabetes	(Tong et al., 2018)
Huang-Lian-Jie-Du decoction (HLJDD)	Parabacteroides, Blautia, Akkermansia, Aerococcus, Staphylococcus-Corynebacterium	SCFAs	Hyperglycemia and insulin resistance	(Chen et al., 2018b)
Qijian mixture	Mainly Bacteroidetes	55 proteins and related metabolism of galactose, valine, leucine, isoleucine, alanine, aspartate and glutamate. Biosynthesis of aminoacyl-tRNA.	Type 2 diabetes	(Gao et al., 2018)
Main component: TCM polysaccharide				
*G*. *lucidum*	Parabacteroides, Roseburia, Eubacterium, Clostridium	ND	Obesity, NAFLD, diabetes mellitus	(Chang et al., 2015)
*H*. *sinensis* mycelium	*P*. *goldsteinii*	ND	Obesity, NAFLD, diabetes mellitus	(Wu et al., 2019)
Mulberry fruit	Bacteroidales, Lactobacillus, Allobaculum, Bacteroides, Akkermansia	ND	Obesity	(Chen et al., 2018a)
*Ganoderma atrum*	ND	SCFAs	Intestinal mucosal dysfunction, type 2 diabetes	(Zhu et al., 2016) (Ying et al., 2020)
Stigma maydis	Lactobacillus, Bacteroides			(Wang et al., 2016)

TCM herbal ingredients	Microbiota affected	Metabolites produced	Target diseases	References
Main component: Proteins/amino acids				
A variety of different herbs	Multiple phyla in microbiota bacteria	-ammonia -amines -gases (methane, hydrogen gas, H_2_S) -catecholamines and phenols (*p*-cresol, *p*-nitrosophenol, *p*-diazoquinone, indoxyl sulfate, hippuric acid, phenyl sulfate, pyrocatechol sulfate, 4-ethylphenyl sulfate, p-cresol glucuronide, and equol 7-glucuronide) -neuro-active metabolites, such as serotonin, melatonin, kynurenine, quinolinate, indole, IAA, IPA, and tryptamine	Chronic inflammation related diseases	(Baumann and Bisping, 1995; Magee et al., 2000; Blachier et al., 2007; Neis et al., 2015; Liu et al., 2016; Mazzoli and Pessione, 2016; Portune et al., 2016; Velenosi et al., 2016; Lin et al., 2017; Ma et al., 2017; Kaur et al., 2019)
Main component: Lipids/fatty acids				
A variety of different herbs	Multiple phyla in microbiota bacteria	-conjugated essential fatty acids (conjugated linoleic acid) -trimethyl amine (TMA)	Chronic inflammation related diseases	(Devillard et al., 2007; Marques et al., 2015; Rath et al., 2017; Janeiro et al., 2018; Schoeler and Caesar, 2019; Yu et al., 2019b)
Main component: Chemicals and related				
A variety of different herbs	Multiple phyla in microbiota bacteria	-secondary glycosides and/or aglycones -CPT-11 related SN-38-glucuronide -secondary bile acids	Chronic inflammation related diseases	(Dabek et al., 2008; Yang et al., 2011; Yan et al., 2013; Chen et al., 2015; Wahlstrom et al., 2016; Ramirez-Perez et al., 2017; Jia et al., 2018)
Ginsenosides in Du-Shen-Tang (DST)	Enhance the growth of *Lactobacillus spp*. and *Bacteroides spp*.	ND	Fatigue, acute cold stress	(Zhou et al., 2016)
Banxia Xiexin decoction (BXD)	ND	Baicalin, baicalein, wogonoside-wogonin, scutellarin, berberine, coptisine, ginsenoside Rb1, ginsenoside Re	Diabetic gastroparesis	(Xu et al., 2018)
Metformin and *Houttuynia cordata* extract (HCE)	*Roseburia*, Akkermansia, Gram-negative bacteria including *Escherichia coli*, *Bacteriodetes fragilis*.	ND	Insulin resistance and metabolic syndromes	(Wang et al., 2017b; Wang et al., 2018a)
Berberine derived from *Coptis chinensis*	Bifidobacterium, *Escherichia coli*	ND	Glucolipid metabolism and insulin resistance in diabetic mice	(Han et al., 2016; Liu et al., 2018b)
Rhein	Bacteroidetes	ND	Antidiabetic effects	(Wang et al., 2018b)

Recent studies have shown a variety of TCM herbal components influence microbial abundance and diversity, which reciprocally is closely related to the efficacy of TCM herbs (Table 1). Among these, effects from PS treatment on obesity, diabetes and related metabolic syndromes seemed to be most intensively studied. For example, treatment with PS purified from *G*. *lucidum* and *H*. *sinensis* mycelium significantly reduced obesity through enhancement of a consortium of commensal bacteria, including *Parabacteroides goldsteinii*, *Roseburia*, *Eubacterium* and *Clostridium spp*. (Chang et al., 2015; Wu et al., 2019). Mulberry fruit PS also influenced obesity and modulated gut microbiota (Chen et al., 2018a), while PS from *Ganoderma atrum* ameliorated intestinal mucosal dysfunction and improved liver function in type 2 diabetes (Zhu et al., 2016), and s*tigma maydis* PS ameliorated type-2 diabetes (Wang et al., 2016) by changing gut microbial populations and related fermentation functions. Further, the PS and ginsenosides contained in decoction of ginseng, the Du-Shen-Tang (DST), restored fatigue and acute cold stress, and modulates the gut microbiota composition (Zhou et al., 2016). There were many other examples highlighting the microbial change during the treatment of obesity, diabetes and metabolic syndromes (Table 1). These examples include Daesiho-Tang (DSHT) that attenuated obesity and significantly increased the relative abundance of Bacteroidetes, B/F ratio, Akkermansia, Bifidobacterium, Lactobacillus, and decreased the level of Firmicutes (Hussain et al., 2016), Gegen Qinlian Decoction (GQD) alleviated Type 2 diabetes and significantly increased *Faecalibacterium prausnitzii* (Xu et al., 2015), while Qushi Huayu Decoction (QHD) reduced HFD-induced non-alcoholic fatty liver disease (NAFLD), and significantly increased the abundance of Parabacteroides and decreased the abundance of Odoribacter, Rikenella, Tyzzerella, Intestinibacter, Romboutsia and 2 members in Lachnospiraceae (Leng et al., 2020). Other examples related to gut microbiota changes included (Table 1): 20% *Folium Mori* amelioration of diabetes (Zhang et al., 2019a), Xiexin Tang-mediated improvement of type 2 diabetes (Wei et al., 2018), amelioration of human type 2 diabetes by metformin and a traditional Chinese herbal formula, AMC (Tong et al., 2018), Huang-Lian-Jie-Du decoction-mediated treatment of hyperglycemia and insulin resistance (Chen et al., 2018b), improvement of type 2 diabetes by treatment with Qijian (Gao et al., 2018), Banxia Xiexin decoction on diabetic gastroparesis rats (Xu et al., 2018), *Houttuynia cordata* facilitation of metformin on reducing insulin resistance (Wang et al., 2017b), berberine, the main bioactive alkaloid of *Coptis chinensis*, on glucolipid metabolism and insulin resistance in diabetic mice (Han et al., 2016; Liu et al., 2018a), and rhein’s role in antidiabetic effects (Wang et al., 2018b). Evidently, efficacy of TCM herbal treatment is closely related to their influence on gut microbiota composition. Therefore, the ingredients of TCM herbs may not only directly regulate host cells activity, but also be metabolized by gut microbiota and affect their structures. Close interaction between TCM and gut microbiota contributes to modulating the host immune and metabolic activities. New research approaches based on gut microbiota-related characterization of clinically applicable TCM components have to be developed (Zhao et al., 2014).

## Role of gut microbiota in fermentation of tcm ps

TCM PS as well as other plant-derived PS such as vegetables, fruits, and whole grains etc. were shown to play important functions in immune-modulation and disease amelioration (Chang, 2002; Yang et al., 2009; Li et al., 2013; Yu et al., 2018b; Sun et al., 2019). For example, both high (>100 kDa.) and low (<100 kDa.) molecular weight, and neutral and acidic PS prepared from *Panax ginseng* that displayed differential functions affecting cellular and host physiology (Sun, 2011; Kim et al., 2020) were among the many examples. Owing to the limited digestive enzymes encoded by the human genome, PS are frequently undigested until they reach the colon (Baumann and Bisping, 1995). In the colon, fermentable PS, such as β-glucans, were broken down by gut microbes via their saccharolytic machinery to produce important groups of natural bioactive products. Serial digestion of PS resulted in production of a number of short-chain oligosaccharides, comprising varying chain lengths, structural conformations, and number of branches (Tzianabos, 2000; Santa et al., 2014). These varied oligosaccharides may function to enhance growth of probiotics, such as *Bifidobacterium spp*. and *Bacteroides spp*. (Bouhnik et al., 2004). The shorter PS may be further digested to form either monosugars such as mannose (Man), glucose (Glc), galactose (Gal), rhamnose (Rha), arabinose (Ara), and fucose (Fuc) which enhance bacterial growth (Koropatkin et al., 2012; Tremaroli and Backhed, 2012). Monosugars can continuously be catabolized to form metabolites such as short chain fatty acids (SCFAs) (formate, acetate, propionate, butyrate), lactate, hydrogen, and carbon dioxide, which may directly affect host physiology (Schwiertz et al., 2010; Simpson and Campbell, 2015; Burokas et al., 2017; Martel et al., 2017a). On the other hand, degraded products such as D-mannose may act as signaling molecules which show differential immunomodulatory effects and functions towards host cells and tissues (Zhang et al., 2017; Zhang et al., 2018).

A consortium of gut bacteria participates in the degradation of TCM-derived PS. For example, a host of bacterial species are involved in butyrate production, including *F*. *prausnitzii*, *Eubacterium rectale*, *Roseburia spp*. *Clostridium spp*. and many others belonging to Bacteroidetes (Zhang et al., 2018). These functional bacteria were characterized by their displays of several to dozens of enzymes used to degrade PS by targeting specific glycosidic linkages or chemical substituents (Martens et al., 2011). Genetic clusters involved in binding, degradation and importation of various PS include miscellaneous polysaccharide utilization loci (PULs), or starch utilization system (Sus)-like systems in Bacteroidetes (Bayer et al., 2008; Ravcheev et al., 2013). Comparatively, with respect to carbohydrate active enzymes (CAZymes), Bacteroidetes degrade a relatively wide range of polysaccharides, while Firmicutes prefer to catabolize selected polysaccharides (Cockburn and Koropatkin, 2016; Zhang et al., 2018). Many different bacterial species may involve sequential catabolism of PS, and the functional metagenomics of consortium of bacteria that participate in PS metabolism are currently under intensive study.

## Tcm affects gut microbiota that produces functional amino acids metabolites

Dietary proteins including those derived from TCM herbs, dietary foods, and other nutraceuticals can be digested by both hosts and gut microbiota, which may further affect gut microbiota structure (Conlon and Bird, 2014; Madsen et al., 2017; Amaretti et al., 2019). At the same time, certain gut bacteria in the distal colon also metabolize amino acids to form unique functional metabolites through specific biochemical pathways (Baumann and Bisping, 1995; Neis et al., 2015; Liu et al., 2016). The metabolic intermediates produced may involve either optimal modulation of energy homeostasis, nutrition metabolism, intestinal health and immunity, or cause inflammation and diseases (Neis et al., 2015; Portune et al., 2016; Lin et al., 2017). Basically, deamination of amino acids results in the production of ammonia, whereas decarboxylation leads to amine production (Baumann and Bisping, 1995). Depending on the amount of proteins up taken, the concentrations of ammonia, trace amines, and gases (methane, hydrogen, H_2_S) related to cytotoxins, genotoxins, and carcinogens produced in colon are altered (Ma et al., 2017). These harmful metabolites may translocate across the intestinal barrier and enter the blood for systematic circulation, resulting diseases development.

Anaerobic fermentation of cysteine and methionine by bacteria results in H_2_S formation. Long term increased sulfide and ammonia concentrations in the colon were shown to promote colitis and tumorigenesis (Baumann and Bisping, 1995; Magee et al., 2000). Also, metabolism of aromatic amino acids may produce a group of uremic toxins, including indoxyl sulfate, p-cresyl sulfate, hippuric acid, phenyl sulfate, pyrocatechol sulfate, 4-ethylphenyl sulfate, p-cresol glucuronide, and equol 7-glucuronide (Velenosi et al., 2016). These compounds are closely related to the development of chronic kidney disease (CKD), where the gut-plasma-kidney metabolic axis is established (Mishima et al., 2017; Liu et al., 2018b). Furthermore, degradation of “tyrosine” gives rise to catecholamines and phenols, where *p*-cresol, *p*-nitrosophenol and *p*-diazoquinone are proposed to show carcinogenic effects (Bone et al., 1976; Kikugawa and Kato, 1988), tyramine is related to hypertension, and DOPA, dopamine and nor-adrenaline can modulate multiple physiological functions (Mazzoli and Pessione, 2016). On the other hand, the anaerobic conversion of “tryptophan” also produces functional metabolites related to the gut-brain axis (GBA). These include neuro-active metabolites, such as serotonin, melatonin, kynurenine, quinolinate, indole, IAA, IPA, and tryptamine (Mazzoli and Pessione, 2016; Kaur et al., 2019). Other neuroactive molecules including histamine, glutamate, and GABA are also synthesized owing to the close interaction between the host and the microbiota (Sharon et al., 2014; Mazzoli and Pessione, 2016). The gut bacteria involved in such metabolism processes were widely categorized into five phyla including Actinobacteria, Firmicutes, Bacteroidetes, Proteobacteria, and Fusobacteria, in which Clostridium, Burkholderia, Streptomyces, Pseudomonas, and Bacillus were further enriched to be involved in the many tryptophan metabolism pathways (Kaur et al., 2019).

Branched chain amino acids (BCAAs), such as leucine (Leu), isoleucine (Ile), and valine (Val), work both as the substrates for anabolism of nitrogenous compounds and as signaling molecules regulating energy homeostasis via multiple signaling networks, including the phosphoinositide 3-kinase/protein kinase B/mammalian target of rapamycin (PI3K/AKT/mTOR) pathway (Nie et al., 2018). Gut microbiota also participates in fermentation of BCAA and generates a complex mixture of metabolites, including ammonia, SCFAs, and branched-chain fatty acids (valerate, isobutyrate, and isovalerate). These bacterial metabolites have been shown to influence normal mucosal immunity of the host (Blachier et al., 2007).

Generally, the abundance of gut microbiota bacteria that are involved in amino acids metabolism are frequently affected by TCM herbs administration (Chang et al., 2015; Lyu et al., 2017; Tong et al., 2018; Feng et al., 2019; Lin et al., 2019; Nie et al., 2019; Wu et al., 2019; Yue et al., 2019; Zhang et al., 2019a). These included the Clostridium clusters, Bacillus, Lactobacillus, Streptococcus, and Proteobacteria in human small intestine, and the Clostridia and Peptostreptococci in large intestine of healthy humans (Neis et al., 2015). More and more bacterial species are expected to be unraveled to be involved in protein metabolism. Therefore, advances with regard to unraveling the protein/amino acid fermentation pathways, the potential novel corresponding metabolites produced by gut microbiota, and modulation of microbiota composition by TCM herbs are essential (Zhang et al., 2019b).

## Tcm-affected gut microbiota produces functional metabolites in lipid metabolism

Lipids and their derived functional metabolites play multiple physiological roles in the host. Lipids from TCM herbs and daily foods intake have also been shown to affect gut microbial growth and composition, while gut microbiota bacteria are also important players in lipid metabolism in hosts (Marques et al., 2015; Di et al., 2019; Schoeler and Caesar, 2019; Yu et al., 2019b) (Table 1). Besides SCFAs, many other functional metabolites derived from lipid metabolism are also produced by bacteria in the gut. For example, commensal bacteria, including Roseburia, Lactobacillus, Butyrivibrio, and Megasphaera participate in fatty acid metabolism to produce an array of conjugated essential fatty acids (e.g., conjugated linoleic acid) that intricately influence host physiology (Devillard et al., 2007). On the other hand, milk fat increases taurine-conjugation of bile acids, which leads to the further growth of *Bilophila wadsworthia* that then uses the increased availability of amino acid-derived sulfur to produce H_2_S closely related to the incidence rates of colitis (Devkota et al., 2012).

Using key enzyme components of the trimethylamine (TMA)-synthesis pathways such as the choline TMA-lyase (CutC) pathway and carnitine oxygenase (CntA) pathway, gut bacteria such as *Clostridium* XIVa strains and *Eubacterium spp*. catabolize choline and l-carnitine to produce TMA. TMA is converted to trimethylamine-*N*-oxide (TMAO) in the liver (Rath et al., 2017; Janeiro et al., 2018). Many studies have indicated a close association between TMAO plasma levels and the risk of atherothrombotic cardiovascular disease (CVD) (Canyelles et al., 2018).

Previous studies have highlighted the roles of gut bacteria, such as Akkermansia, Butyricimonas, Christensenellaceae, Eggerthella, Tenericutes, and Pasteurellaceae, on affecting specific aspects of lipid metabolism and/or distinct classes of lipoproteins (Ghazalpour et al., 2016). At the same time, rapid progresses have been made in unraveling the underlying mechanisms of TCM-mediated regulation of lipid metabolism and amelioration of disease. For instance, TCMs have been shown to inhibit intestinal absorption, reduce de novo biosynthesis, increase catabolism, and enhance secretion in lipids (Bei et al., 2012). The changed gut microbiota composition by TCM herbs administration may contribute to the ameliorative effects on abnormal lipids metabolism (Huang et al., 2019; Zhang et al., 2019a). On the other hand, ginseng extract can enrich *Enterococcus faecalis* that produces an unsaturated long chain fatty acid, myristoleic acid (MA), leading to reducing adiposity by activation of brown adipose tissue (BAT) and formation of beige fat (Quan et al., 2019). Therefore, modulation of gut microbiota bacteria by TCM herbs may produce optimal amounts of beneficial lipid metabolites, which is an effective strategy for promotion of well-being in hosts.

## Transformation of tcm small chemical molecules by gut microbiota

Different TCM formulae contain an array of small chemical molecules with a number biological functions, including flavonoids, saponins, alkaloids, and anthraquinones, etc. (Xu et al., 2013; Yan et al., 2013; Chen et al., 2015; Zhang et al., 2020b) (Table 1). Both gut microbiota and the liver involve conversion of the xenobiotics ingredients into subsequent metabolites (Fig. 1). In the intestines, these components can be modified/deconjugated by gut microbiota. Alternatively, they can also be absorbed and transported to the liver, where they are also modified/conjugated to increase their water solubility and facilitate excretion. After excretion into the intestinal tract from liver, these metabolites may further undergo modification/deconjugation by gut microbiota to form secondary metabolites (Wahlstrom et al., 2016; Kim, 2018). Many of the transformed metabolites frequently act as functional compounds directly influencing the curative effects of TCM treatment. For example, herbal glycosides such as saponins, geniposide, iridoid glycosides, and flavone glycosides frequently identified in TCM ingredients are metabolized into secondary glycosides or aglycones by bacterial β-glucosidase (GUS) (Dabek et al., 2008; Yang et al., 2011) (Fig. 1). Such transformation may affect the bioavailability and bioactivity of these molecules (Yang et al., 2011). Additionally, the gut microbial GUS enzymes encoded by a variety of gut bacteria belonging to Firmicutes (60%) and Bacteroidetes (21%) (Humblot et al., 2007; Creekmore et al., 2019) also catalyze hydrolysis of β-D-glucuronic acid from their conjugated compounds and influence drug potency and toxicity. The transformation of ginseng extracts is taken as an example (Fig. 1). After oral administration, ginsenosides such as Rb1, Rb2 and Rc from ginseng extracts were transformed by gut microbiota to form 20-*O*-β-D-glucopyranosyl-20(*S*)-protopanaxadiol (compound K) which was absorbed into the blood to achieve the pharmacological functions. Compound K showed more potent anti-tumor, anti-inflammatory, and anti-allergic activities more than ginsenoside Rb1 (Wang et al., 2011; Kim et al., 2013). Therefore, the gut microbiota plays an important role in the pharmacological action of orally administered ginseng.

Another example is the CPT-11 which is a potent anticancer agent metabolized to the active compound, SN-38 *in vivo*. A balanced SN-38 concentration in patients is essential to maintain optimal efficacy of cancer treatment while reducing toxicity. Intriguingly, both TCM components and gut microbiota are involved in modulation of SN-38 activity. For detoxification, SN-38 is conjugated to SN-38-glucuronide by UDP-glucuronosyltransferase (UGT). However, purified herbal aglycons where the glycosyl group of a glycoside was removed inhibited UGT activity (Yokoi et al., 1995; Ramesh et al., 2010; Bailly, 2019), leading to an increase in toxic SN-38 concentration in the enterohepatic circulation. On the other hand, gut microbiota bacteria also use the *gus* encoded β-glucuronidase for deconjugation of the SN-38-glucuronide. Therefore, the underlying regulatory activities formed a complicated regulatory network (Dabek et al., 2008). How to achieve a balanced SN-38 activity in patients with maximal efficacy of cancer treatment while reducing toxicity is an important issue.

Gut microbiota also transform bile acids and cholesterols, producing a variety of functional metabolites (Gerard, 2013). Among these, primary bile acids are produced in the liver as glycine, taurine, or sulfate conjugates, and after secretion into the intestine, are deconjugated and modified by intestinal bacteria. Through interaction with bile acid farnesoid X receptor (FXR) and G protein-coupled bile acid receptor 1 (TGR5), bile acids signaling controls multiple important physiological behaviors and maintains intestinal homeostasis and a healthy environment (Jia et al., 2018). Bacteria such as Bacteroides, Bifidobacterium, Clostridium, Egghertella, Escherichia, Eubacterium, Fusobacterium, Lactobacillus, Listeria, Peptococcus, Peptostreptococcus, Pseudomonas, and Ruminococcus, use their enzymes in deconjugation, oxidation and epimerization, 7-dehydroxylation, esterification, and desulfatation of the bile acids (Gerard, 2013). Secondary bile acids that might work as tumor promoters are therefore produced after bacterial fermentation in intestine (Wahlstrom et al., 2016; Ramirez-Perez et al., 2017; Jia et al., 2018). Accordingly, the development of disease-treatment strategies using TCM herbs, or use of metabolically engineered bacteria to modify chemicals for maintaining health are warranted (van Duynhoven et al., 2011; Lee et al., 2012; Anlu et al., 2019).

## Microbiota-based integrated multiomics study-the next generation tcm herbal research

**Figure 2:**
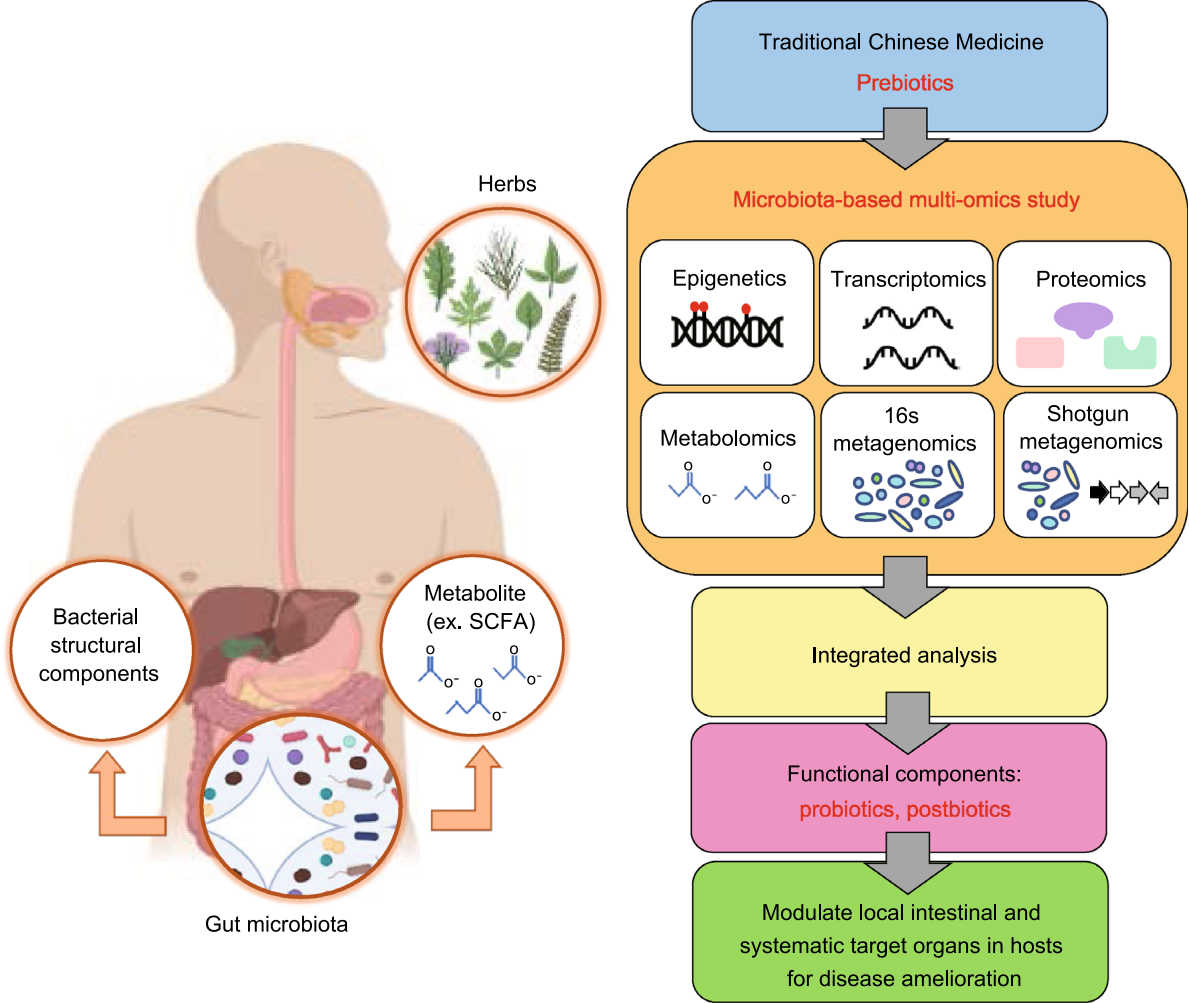
Microbiota-based integrated multi-omics platforms for TCM-derived herb study. The basic rationale is to identify TCM herbs related active components after transformation by gut microbiota fermentation. The multiomics platforms such as, epigenetics, 16s metagenomics, shotgun metagenomics, transcriptomics, proteomics, and metabolomics are to be used, followed by the integrated analysis. Such strategy may identify novel TCM prebiotics, bacteria (probiotics) and metabolites (postbiotics) as well as bacterial structural components (paraprobiotics) to modulate local intestine and systematic target organs in hosts for disease amelioration

Owing to the difficulties encountered in TCM research, one must consider developing novel strategy to characterize TCM herbs related active components. For future TCM-derived herbal studies, use of microbiota-based integrated multiomics platforms seems critical (Fig. 2). The underlying basic rationale is as follows: instead of directly screening functional components from herbal extracts, identifying functional components after transformation by gut microbiota fermentation using multiomics approaches (Fig. 2). Prepared TCM herbal products (such as crude extracts/decoctions or powders), are first fed to animals to evaluate their efficacy of disease amelioration. If positive results are obtained, the classification and abundance of bacteria in intestine/feces, and their derived metabolites (including bacterial structural components containing potential paraprobiotics) in intestine/feces/blood samples are subsequently quantified. Bacteria and metabolites that show statistical differences between control and experimental groups may be involved in treatment efficacy of the disease in question, and accordingly will be targets of interest. These bacteria (potential probiotics) or compounds (potential postbiotics or paraprobiotics), either singly or in a consortium, will then be systematically assessed for their function and mechanism. To achieve this, cutting-edge analyses platforms, such as next generation sequencing (NGS), proteomics, and metabolomics are to be used.

The experimental design of microbiota-based TCM multiomics research is described as follows (Fig. 2): (i) In preparing TCM herbal products such as decoction, strictly follow the instructions for consistency. (ii) Optimally grouping the animals and humans, followed by observing disease progression and development, and finally evaluating the results by statistics. (iii) The composition of gut microbiota and/or their predicted functions of gut microbiome are analyzed by 16S rRNA gene sequencing, or shotgun sequencing, respectively, followed by bioinformatics analyses on operational taxonomic units (OTUs), and other functional DNA fragments. (iv) Metabolites will be analyzed by metabolomics combined with other analyses such as metatranscriptomics (for gut microbiota), and epigenetics, transcriptomics/single cell RNA sequencing (scRNA seq), and proteomics (for host). This multiomics approach will work in concert with traditional immunological, physiological, and pathological analyses for holistic results. (v) Associations between and among datasets obtained from control and experimental groups are analyzed to identify parameters with significant differences. Potential enriched or decreased biochemical pathways are established by GO and KEGG pathway analyses. Besides, some databases related to TCM and gut microbiota research including the TCM-Mesh, TCMSP, Traditional Chinese Medicines Integrated Database (TCMID), Compound Reference Database (CRD) and HIT, as well as pathogen-host databases (PHI-base and EHFPI) can be used as references of analytical systems for TCM pharmacology network analysis (Zhang et al., 2020a, b). (v) Isolate selected microbiota bacteria and purify or synthesize novel functional metabolites for further evaluation or validation of their safety and efficacy. (vii) Based on the results obtained, evaluate the possibility of further clinical trials.

Critically, after fermentation of TCM ingredients by microbiota, only important elements involved in the regulation of signaling pathways and diseases amelioration are highlighted. These elements are then selected to develop new treatment strategies. Currently, there are many multiomics-based templates that can be recruited as references for use in TCM and microbiota related studies. For instance, a functional analysis of the gut microbiota ecosystem for better understanding inflammatory bowel disease (IBD) (Lloyd-Price et al., 2019), while the modified ColPortal platform (Esteban-Gil et al., 2019) integrates multiomics studies to understand the relationship between the microbiota and metabolomics in inflammatory bowel disease (IBS) disease model (Liu et al., 2019b). The pipelines described here have the potential to identify novel TCM-based herbal prebiotics, probiotics, and postbiotics for treatment of disease.

## Perspective

Recent studies have demonstrated that gut microbiota participate in the metabolism of foods and nutrients and play central roles in the transformation of original TCM herbal components into functional metabolites. Under increasing studies on characterizing the metabolites after administration of various TCM herbs and gut microbiota transformation, together with the help from cutting-edge multiomics research tools, such as the NGS and metabolomics platforms, as well as the advanced bioinformatics analyses, databases, and algorithms, the identification of novel and effective metabolites for safe treatments is possible. Ultimately, a better understanding of the molecular mechanisms TCM function will make great contributions to the effective treatment of the chronic inflammation related diseases.

## Abbreviations

ADME: absorption/distribution/metabolism/excretion
Ara: arabinose
BAT: brown adipose tissue
BCAAs: branched chain amino acids
CAZymes: carbohydrate active enzymes
CFU: colony-forming units
CKD: chronic kidney disease
CRD: compound Reference Database
C-T-D: component-target-disease
CVD: cardiovascular disease
DAMPS: damage-associated molecular pattern molecules
DSHT: Daesiho-Tang
DST: Du-Shen-Tang
F/B: Firmicutes/Bacteroidetes
Fuc: Fucose
FZHY: Fuzheng Huayu
Gal: galactose
GI: gastrointestinal
Glc: glucose
GO: Gene Ontology
GQD: Gegen Qinlian decoction
GUS: β-glucosidase
HTS: high-throughput screening
IBD: inflammatory bowel disease
IECs: intestinal epithelial cells
Ile: isoleucine
KEGG: Kyoto Encyclopedia of Genes and Genomes
Leu: leucine
MA: myristoleic acid
Man: mannose
NAFLD: non-alcoholic fatty liver disease
NGS: next generation sequencing
OTUs: operational taxonomic units
PAMPS: pathogen-associated molecular pattern molecules
PD: pharmacodynamics
PK: pharmacokinetics
PS: polysaccharides
PULs: polysaccharide utilization loci
QHD: Qushi Huayu Decoction
Rha: rhamnose
SCFAs: short chain fatty acids
scRNA seq: single cell RNA sequencing
Sus: starch utilization system
TCM: Traditional Chinese Medicine
TCMID: Traditional Chinese Medicines Integrated Database
TCMSP: TCM System Pharmacology Database and Analysis Platform
TMA: trimethylamine
TMAO: trimethylamine-*N*-oxide
T-P: target-pathway
UGT: UDP-glucuronosyltransferase
Val: valine
